# Supplementary material for: Identifying healthcare needs with patient experience reviews using ChatGPT
Source: PLoS One. 2025 Mar 18;20(3):e0313442. doi: 10.1371/journal.pone.0313442 (PMC11918364; doi:10.1371/journal.pone.0313442)
Supplement: Attachment — Submitted filename: comments.docx [file pone.0313442.s001.docx]

This work used the reviews to create Aspect-Based Sentiment Analysis (ABSA) templates, categorizing patient reviews into three categories that reflect patients' areas of concern. By introducing thought chains, the ABSA templates were embedded into prompts for ChatGPT, which was then used to identify patient needs. The results show that the proposed method achieved a weighted total precision of 0.907, which is outstanding compared to the direct narrative tasks in ChatGPT.

Overall, this work is complete, but there is still room for improvement. My suggestions are as follows:

1. Lines 53-54 “but these techniques still struggle to pinpoint specific areas of patient dissatisfaction within individual reviews” : is there any literature to support this statement?
2. Lines 51 - 59：In this paragraph, the first part ("Recent research... individual reviews") discusses the limitations of deep learning and data mining approaches in analyzing patient reviews, but the second part ("Finally, some studies... patient experience") covers other topics. These two parts lack a clear logical connection. It is recommended to revise this paragraph to ensure clearer and more coherent logic.
3. Lines 112 -123 : The hyperparameters within ChatGPT are set to their default values. Is it possible to ensure that the parameters used are optimal without tuning them? Could the model's performance be improved by adjusting these hyperparameters?
4. The test size is 2000, out of a total data size of 504,198. This is a relatively small proportion (approximately 0.4%) for the test set, which might not provide a robust estimate of the model's performance on unseen data.
5. Lines 245- 246: For the test set (2000 samples), labeling was done manually. For larger datasets, relying on manual labeling is very inefficient. Are there any better methods to improve efficiency?
6. It is recommended to add an explanation of how the three metrics (Precision, Recall, and F1-weight) are used to evaluate the model's performance. For example, the higher the Precision, the better the model's performance.
7. Does the F1 score in Table 3 represent the weighted F1 score ? And is the F1 score in the *Model Performance* section also referring to the weighted F1 score? The authors should provide a clear description.
